# Assessing Emotional and Social Intelligence in Portuguese Youth: Structural Validity of the EQ-i:YV

**DOI:** 10.3390/ejihpe16090139

**Published:** 2026-09-13

**Authors:** Adelinda Araújo Candeias

**Affiliations:** Department of Medical Sciences and Health, Comprehensive Health Research Centre (CHRC), University of Evora, Largp dos Colegiais, Ap. 94, 7005-554 Evora, Portugal; aac@uevora.pt

**Keywords:** emotional intelligence, EQ-i:YV, children, adolescents, confirmatory factor analysis, psychological assessment

## Abstract

The Emotional Quotient Inventory: Youth Version (EQ-i:YV) assesses how young people perceive their emotions, relationships, coping with pressure, adaptability, and general mood. We evaluated whether its five domains provide a coherent yet differentiated representation of emotional-social functioning in 931 Portuguese participants aged 8–16 years. Responses to 54 substantive items were analysed as ordered categories in stratified exploratory and confirmatory subsamples. A restrictive five-factor confirmatory factor analysis did not adequately represent the response pattern, whereas a five-domain Target-ESEM that allowed small associations with related competencies provided a better and internally replicated representation. Domain reliability was adequate to strong (ordinal α = 0.744–0.865; ω = 0.760–0.866). Although the overall composite was reliable, variance attributable to one general factor was only moderate (ωH = 0.592), and the higher-order solution was unstable. The Portuguese EQ-i:YV should therefore be interpreted primarily through its five-domain profile. A global score, when required for descriptive continuity, should be presented only as a secondary multidimensional summary alongside the domain scores.

## 1. Introduction

### 1.1. Emotional Intelligence: Competing Models and Measurement

Emotional intelligence (EI) refers broadly to differences in how people notice, understand, use, and regulate emotional information. In everyday life, these processes shape how a young person makes sense of inner experience, responds to other people, copes with frustration, and adjusts behaviour when circumstances change. They may be especially important during childhood and adolescence, when self-awareness, identity, peer relationships, and the capacity to regulate emotion are still developing. Emotional competencies during these years have been associated with psychological well-being, social functioning, school adjustment, and academic achievement (Calero et al., 2018; Franco et al., 2017).

The field has not yet reached a single, shared understanding of what emotional intelligence is or how it should be assessed. Ability models view emotional intelligence as a set of mental abilities and usually assess how well a person performs on tasks with answers evaluated against predefined criteria (Mayer et al., 2008). Trait models take a different perspective, focusing on how people generally perceive and describe their own emotional experiences and tendencies through self-report questionnaires. Mixed models adopt a broader view, bringing these self-perceptions together with social competencies, motivation, coping resources, and the capacity to adapt to everyday demands (Bar-On, 2006; Bru-Luna et al., 2021). Although these approaches share some common ground, they describe and assess different aspects of emotional functioning. Their scores must therefore be interpreted in relation to the psychological construct being examined, the way participants are asked to respond, and the assessment method used.

Research has not yet reached a consensus on how emotional intelligence should be conceptualized or assessed. Ability models understand emotional intelligence as a set of mental abilities and generally assess performance through tasks evaluated against predefined criteria (Mayer et al., 2008). Trait models adopt a different perspective, focusing on how people typically perceive and describe their own emotional experiences and tendencies through self-report questionnaires. Mixed models offer a broader understanding by integrating these self-perceptions with social competencies, motivation, coping resources, and the capacity to adapt to everyday demands (Bar-On, 2006; Bru-Luna et al., 2021). Although these approaches share some conceptual ground, they represent different aspects of emotional functioning and should not be treated as interchangeable. The meaning of any score must therefore be considered in relation to the psychological construct being assessed, the way participants are asked to respond, and the assessment method used.

Current research does not provide a consensual definition of emotional intelligence or a unified approach to its assessment. Ability models conceptualize emotional intelligence as a set of mental abilities and generally assess performance through tasks evaluated against predefined criteria (Mayer et al., 2008). Trait models adopt a different perspective, focusing on how individuals typically perceive and describe their own emotional experiences and tendencies through self-report questionnaires. Mixed models offer a broader conceptualization by integrating these self-perceptions with social competencies, motivation, coping resources, and adaptive functioning (Bar-On, 2006; Bru-Luna et al., 2021). Although these approaches share some conceptual ground, they address distinct aspects of emotional functioning and should not be treated as interchangeable. The interpretation of any score must therefore remain closely aligned with the psychological construct being assessed, the response process through which the score is obtained, and the assessment method employed.

Within the mixed-model tradition, Bar-On described emotional-social intelligence as a network of related competencies, skills, and facilitators that help people understand and express themselves, relate to others, manage everyday demands, solve personal and interpersonal problems, and maintain a constructive outlook (Bar-On, 2006). The model distinguishes five broad components: Intrapersonal, Interpersonal, Stress Management, Adaptability, and General Mood. General Mood is understood mainly as a resource that supports emotionally and socially effective behaviour, rather than as a narrow emotional ability. Positive Impression serves a different purpose: it flags a possibly over-favourable presentation of the self and is therefore a response-style indicator, not a substantive EI domain. The theory thus anticipates five connected but distinguishable areas and permits a broad composite; it does not require every response to reflect one perfectly isolated trait.

The distinction between perceived and performance-based EI is especially important when assessing children and adolescents. An EQ-i:YV response tells us how a young person sees their typical emotional and social functioning; it does not show how that person would perform on a task with objectively correct answers. Such self-descriptions are shaped by emotional competence, but also by reading comprehension, developing self-knowledge, social comparison, local expectations, and willingness to disclose personal experience. They can therefore illuminate how a young person understands themselves without becoming a direct test of emotional problem-solving. Associations with self-esteem, self-concept, social competence, well-being, and school adjustment support the wider relevance of perceived EI while also showing its overlap with other self-evaluative and personality-related processes (Calero et al., 2018; Franco et al., 2017).

For this reason, internal structure is only one part of the evidence needed to interpret an EI score. Responsible use also depends on understanding how young people arrive at their responses and how the scores relate to other variables, including convergent, discriminant, criterion-related, predictive, and incremental evidence. Structural validity remains a necessary starting point: links with school adjustment, well-being, or mental-health criteria are difficult to understand if the score does not represent the intended domains. This study therefore concentrates on internal structure, reliability, and their consequences for interpretation, while not claiming to provide every form of validity evidence.

### 1.2. The EQ-i:YV and Its International and Portuguese Evidence

The Emotional Quotient Inventory: Youth Version (EQ-i:YV) operationalizes the Bar-On framework for the assessment of emotional-social intelligence in children and adolescents (Bar-On & Parker, 2000). The full form comprises 60 self-report items answered on a four-category response scale. Of these, 54 substantive items assess five domains—Intrapersonal, Interpersonal, Stress Management, Adaptability, and General Mood—whereas the remaining six items constitute the Positive Impression scale. The 30-item short form focuses on four core domains and does not include General Mood or Positive Impression within its substantive structure. Evidence obtained from the full and short forms may therefore be complementary, but the two versions should not be regarded as tests of identical measurement models.

International research on the full EQ-i has generally supported its multidimensional organization, although the allocation and performance of individual items have varied across cultures, samples, and analytical approaches. Ferrándiz et al. (2012) examined the instrument in 1655 Spanish students and identified a five-component structure after excluding the Positive Impression items. Internal consistency coefficients for the domains ranged from 0.63 to 0.80, although items 3 and 39 showed particularly weak item–total relationships. The study also examined associations with intelligence, personality, self-concept, and academic achievement. In a sample of 386 gifted Spanish adolescents, Sáinz et al. (2014) similarly identified five components, which explained 41.1% of the variance, with reliability coefficients exceeding 0.75. These findings broadly supported the organization proposed by Bar-On while also indicating variability in item functioning. However, both studies relied predominantly on exploratory component or factor-analytic approaches and did not examine the instrument through ordinal structural models that allow for small but meaningful cross-loadings.

Research involving shorter forms further shows that an identical structure cannot be assumed across versions and populations. Navarro-Roldán et al. (2023) evaluated the Spanish short form in Colombian children and adolescents aged 8–14 years, dividing the sample for exploratory and confirmatory analyses. Analyses were conducted using JASP 0.12, SPSS 28, and AMOS 24.0. The best-fitting solution comprised four factors and 21 items (CFI = 0.951, TLI = 0.944, RMSEA = 0.030), with McDonald’s omega coefficients ranging from 0.57 to 0.75. More recently, Henning et al. (2026) evaluated a 30-item, four-factor short form and reported acceptable confirmatory model fit, measurement invariance across sex and educational level, satisfactory internal consistency and six-month test–retest reliability, and meaningful associations with alexithymia. Collectively, these findings support the differentiation of emotional-social domains but also demonstrate that item configurations and measurement structures may vary according to the instrument version, population, and analytical method.

The research version of the EQ-i:YV used in the present study was developed and progressively evaluated through a series of translation, cultural adaptation, and psychometric studies involving Portuguese children and adolescents (Candeias & Rebocho, 2007; Prieto et al., 2009; Candeias et al., 2011, 2013). Of particular relevance is a study conducted with children and adolescents aged 8–17 years, in which the global score showed good internal consistency (α = 0.87), while coefficients for the specific scales ranged from 0.69 to 0.86. Exploratory factor analysis identified five main dimensions, which together explained 50.6% of the total item variance. Internal consistency was generally adequate across these dimensions, although a lower coefficient was obtained for Stress Management (α = 0.60; Candeias et al., 2011). These early findings provided encouraging support for the multidimensional structure of the European Portuguese research version while also identifying aspects requiring more detailed psychometric examination.

Portuguese research has also shown that emotion understanding and social-emotional competence are meaningfully related to school achievement (Franco et al., 2017). Evidence concerning developmental patterns is less uniform. In a Spanish study combining longitudinal and cross-sectional data from adolescents, Esnaola et al. (2017) found no substantial age-related change in most short-form dimensions, apart from Stress Management among girls. Building on the earlier adaptation and exploratory studies of the Portuguese EQ-i:YV (Candeias & Rebocho, 2007; Candeias et al., 2011, 2013), Candeias (2025) examined differences in perceived emotional intelligence according to age and sex among Portuguese children and adolescents. The findings did not reveal a uniform developmental pattern across the five domains. Perceived Adaptability and Intrapersonal functioning tended to decrease with age, whereas Interpersonal functioning and Stress Management remained comparatively stable. Interpersonal functioning was the most strongly perceived domain, while Intrapersonal functioning was the least strongly perceived. Some sex-related differences also emerged at particular developmental stages: girls reported higher Interpersonal functioning and Stress Management in early adolescence, whereas boys reported a more positive General Mood in middle adolescence.

These differences were specific to particular domains and developmental stages and were not reflected in significant differences in the global EQ-i:YV score. This raised an important question for the interpretation of the instrument: does the global score conceal meaningful differences in how young people perceive their emotional and social functioning? Although previous Portuguese studies had provided encouraging exploratory evidence for a five-domain structure (Candeias et al., 2011, 2013), a more detailed examination of the item-level structure was still needed to determine whether these domains remained empirically distinguishable, whether their organization was stable, and whether a single global score adequately represented the response pattern. These questions provided the main rationale for the present study.

### 1.3. Capturing the Complexity of Emotional-Social Functioning

The EQ-i:YV is intended for a wide developmental period, from childhood to late adolescence. Young people at these different stages do not necessarily understand themselves, their emotions, or their relationships in the same way. Across these years, they experience important changes in cognitive development, emotional self-awareness, social comparison, interpersonal relationships, and identity formation (Crone & Dahl, 2012; Meeus, 2011). Experiences associated with sex and gender-related social expectations may further influence how they understand and describe their emotional functioning. Using the same instrument across this age range offers valuable continuity, but it also requires sensitivity to developmental differences in how young people interpret the items and use the response options.

Each EQ-i:YV item provides four ordered response options indicating how closely a feeling or behaviour describes the respondent. Although these categories follow a meaningful order, the psychological distance between adjacent options cannot be assumed to be equal. Responses may also be concentrated in the higher categories, particularly for positively worded behaviours and self-perceptions. Analysing them as if they were continuous and normally distributed could therefore misrepresent the relationships among items and the fit of the measurement model (Flora & Curran, 2004; Rhemtulla et al., 2012).

Ordinal methods more closely reflect how these responses are structured. Polychoric correlations estimate the associations between the underlying response tendencies represented by the observed categories, while robust weighted least-squares estimation accommodates both their ordinal nature and potentially asymmetric distributions (Flora & Curran, 2004; Li, 2016; Savalei, 2021). Treating the items as ordinal is therefore more than a technical choice: it brings the analysis closer to the way children and adolescents actually respond to the questionnaire. However, response categories selected by very few participants can make estimation less stable, particularly when groups are compared, and therefore require careful examination (Millsap & Yun-Tein, 2004; Wu & Estabrook, 2016).

A second challenge arises from the close relationship among the five EQ-i:YV domains. In everyday life, emotional and social competencies do not operate in separate psychological compartments. Dealing with a difficult situation, for example, may require a young person to recognize their own emotions, understand another person’s perspective, manage stress, and adjust their response. Independent-clusters confirmatory factor analysis (ICM-CFA) provides a strict test of the proposed structure by requiring each item to reflect only its designated domain. For competencies that naturally work together, however, requiring every association with neighbouring domains to be exactly zero may be too restrictive. Even small cross-loadings can reduce model fit and artificially increase the estimated correlations among factors (Asparouhov & Muthén, 2009; Marsh et al., 2014).

Target exploratory structural equation modelling (Target-ESEM) provides a more flexible but still theory-guided approach. Each item is expected to relate most strongly to its intended domain, while smaller relationships with conceptually related domains are also estimated. Comparing ICM-CFA with Target-ESEM can therefore help clarify whether poor CFA fit reflects a failure of the proposed five-domain structure or a more realistic degree of overlap among related emotional-social competencies.

The meaning of the global score also requires careful consideration. Strong relationships among domains and high overall reliability do not necessarily mean that the instrument measures one homogeneous characteristic. Two young people with similar global scores may have quite different patterns of interpersonal confidence, emotional self-awareness, stress management, adaptability, and mood. A single total score may conceal these differences. Omega hierarchical helps to address this issue by estimating how much of the reliable variance in the total score can be attributed to one general factor after domain-specific variance has been considered. It is therefore more informative about the justification for a global score than coefficient alpha or omega total alone (Reise et al., 2013; Rodriguez et al., 2016).

Finally, internal structure is only one part of the evidence needed to support score interpretation. Contemporary testing standards understand validity as an accumulating body of evidence concerning both the meaning of scores and their intended uses (American Educational Research Association et al., 2014). A broader validation programme for the EQ-i:YV should therefore examine how its scores relate to other emotional and social constructs, how well they remain distinct from different constructs, and whether they are associated with relevant behavioural, educational, or mental-health outcomes. The present study focuses on internal structure and score reliability, supported by complementary convergent and discriminant diagnostics. It does not, however, provide a complete examination of criterion-related, predictive, or incremental validity.

### 1.4. Aim of the Present Study

The present study offers a more detailed and complementary psychometric evaluation of the Portuguese EQ-i:YV. It builds on the initial exploratory factor analysis reported by Candeias et al. (2011) and on our later investigation of age- and sex-related differences in perceived emotional intelligence, which was based on the same participant sample used in the current study (Candeias, 2025). Whereas the earlier studies focused on the instrument’s exploratory structure and on differences between groups, the present study considers more closely how the EQ-i:YV is structured and how its domain and global scores should be interpreted.

This question emerged naturally from our previous findings. Candeias (2025) identified age- and sex-related differences in specific emotional-social domains, although these patterns were not reflected in significant differences in the global EQ-i:YV score. This distinction matters psychologically. Two young people with similar global scores may perceive their emotional and social functioning in quite different ways. One may feel confident in relationships but struggle to understand and express personal emotions, whereas another may manage stress effectively but find it difficult to adapt when circumstances change. These findings therefore raised an important psychological and psychometric question: does the EQ-i:YV adequately preserve meaningful differences across emotional-social domains, or might these differences be obscured when they are summarised in a single global score?

Against this background, the present study sought to establish whether the five intended domains provide a coherent yet differentiated account of how children and adolescents perceive their emotional and social functioning. At the same time, the analyses allowed for the natural overlap among competencies that frequently operate together in everyday life.

More specifically, we assessed whether the five-domain structure could be replicated across two stratified subsamples, whether items were primarily associated with their intended domains, and whether each domain provided sufficiently reliable information to support interpretation. We also considered whether the evidence justified the interpretation of a global score and conducted a cautious, provisional assessment of whether the measurement structure showed broadly comparable patterns for girls and boys. To address these questions, correlated five-factor and second-order CFA models were compared with a five-factor Target-ESEM model. These analyses were followed by internal cross-validation, an assessment of item-level stability, and estimates of domain- and global-score reliability. The purpose was not merely to identify the model with the best statistical fit, but to understand what the resulting scores can meaningfully tell us about young people’s emotional-social functioning—and where a more cautious interpretation is required.

## 2. Materials and Methods

### 2.1. Participants

The analytic sample comprised 931 Portuguese students aged 8–16 years, including 495 girls and 436 boys. Participants attended basic or secondary education in 15 schools located in northern, central, and southern Portugal. The participating institutions included public schools as well as private and cooperative schools. Data were collected within a broader project monitoring the psychological development of children and adolescents aged 8–18 years; the sample available for the present study covered the 8–16-year age range. Table 1 presents the distribution of participants by age and sex.

Schools participated through collaboration with the research team, and recruitment was therefore based on convenience rather than probability sampling. Despite its geographical and educational diversity, the sample should not be regarded as nationally representative. Convenience recruitment can alter the distribution of developmental and contextual characteristics relevant to item responses and can therefore limit the stability and transportability of estimated latent structures (Bornstein et al., 2013; Simons et al., 2017). To protect underage participants’ privacy in the school context, only the sociodemographic information required for this study was retained; socioeconomic and family characteristics were therefore unavailable for analysis. Accordingly, the present factorial findings are evidence for this geographically varied school sample, not population norms or nationally representative estimates.

The present sample also provided the basis for an earlier study by Candeias (2025), which explored differences in perceived emotional intelligence according to age and sex among Portuguese children and adolescents. Although previous Portuguese studies had already provided evidence supporting the validity of the EQ-i:YV (Candeias et al., 2011, 2013; Pires et al., 2012), the findings of the 2025 study highlighted new questions about the psychometric structure underlying these individual differences and about how confidently the domain and global scores could be interpreted. The present study was developed to explore these questions more closely by examining the ordinal structure of the 54 substantive items, the reliability and replicability of the factor solution, and the interpretation of the domain and global scores. The item-level CFA and Target-ESEM analyses presented here therefore extend the earlier research and were not included in the previous publication.

### 2.2. Instrument

The Emotional Quotient Inventory: Youth Version (EQ-i:YV; Bar-On & Parker, 2000) is a 60-item self-report questionnaire designed to assess how children and adolescents perceive their usual emotional and social functioning. Responses are given on a four-point ordered scale ranging from 1 (very seldom true of me) to 4 (very often true of me). After reverse-scoring the relevant items, higher scores indicate a stronger self-perception of the emotional-social competency being assessed.

Fifty-four items represent five substantive domains. Intrapersonal functioning (6 items) concerns recognizing and expressing one’s own emotions. Interpersonal functioning (12 items) covers empathy, social understanding, and relationships with others. Stress Management (12 items) reflects the perceived ability to cope with emotional pressure and regulate impulsive reactions. Adaptability (10 items) concerns flexible thinking and problem-solving when circumstances change. General Mood (14 items) encompasses optimism and a broadly positive outlook. The remaining six items form the Positive Impression scale, which helps identify a potentially over-favourable presentation of the self.

The present study used a version developed by Candeias and Rebocho (2007) for scientific and academic purposes in Portugal. Given the linguistic and cultural proximity between Portugal and Spain, the adaptation process considered the preliminary Spanish materials and associated research (Ferrándiz et al., 2006). Each item was also compared with the original English instrument to preserve the psychological meaning intended by Bar-On and Parker (2000). Translation, back-translation, and linguistic and cultural review were supported by Portuguese–Spanish and Portuguese–English bilingual specialists and followed the test-adaptation principles available from the International Test Commission at the time (International Test Commission, 2005). Particular attention was given to wording that sounded natural and was understandable to Portuguese children and adolescents. The resulting version was subsequently tested and refined through pilot work in Portuguese schools and successive psychometric studies (Candeias et al., 2011, 2013; Pires et al., 2012).

Earlier Portuguese research provided preliminary support for the five-dimensional organization of the instrument, with the five factors explaining 50.6% of the item variance. The global score showed good internal consistency (α = 0.87), while reliability was generally adequate across the specific dimensions, although lower for Stress Management (Candeias et al., 2011). This evidence provided the empirical basis for the more detailed examination undertaken in the present study.

Items were scored according to the predefined scoring key, including reverse scoring where required. Because Positive Impression primarily provides information about response style rather than representing a substantive emotional-social domain, its six items were retained as part of the questionnaire but excluded from the structural models examined in this study. The numerical scoring key used in the analyses is documented in Supplementary Table S10 without reproducing copyrighted item wording.

### 2.3. Procedure and Ethics

Data were collected between January and April 2023 during a later stage of the RED—School Performance and Development project (PTDC/CPE-CED/104884/2008), coordinated by the University of Évora and funded by the Portuguese Foundation for Science and Technology. The work later continued within the complementary REFLECT, COMPUSEL Erasmus+ frameworks and INOVA CJ+, with UIDP/04923 as the aggregating research unit. Schools involved in the original RED project were not recruited again. Trained researchers met students in their schools and administered the questionnaires in group sessions lasting about 30 min. Participation was authorized by the Portuguese Data Protection Commission, the Ministry of Education’s Committee for Monitoring Studies in School Settings (MIME approval no. 0449600001), and the directors of participating schools and institutions. Parents or legal guardians gave informed consent. Students received age-appropriate explanations of the purpose of the study, the confidentiality and anonymity of their answers, the voluntary nature of participation, and their freedom to stop at any point without penalty.

### 2.4. Data Preparation

Before modelling, we checked the data item by item and participant by participant. We verified that responses used the permitted categories (1–4), that scoring direction was correct, and that invalid codes and missing responses were identified. We also examined each item’s response frequency, skewness, and kurtosis. Values outside the prespecified coding rules were treated as missing, apart from one correction verified against the source. Because responses were bounded categories rather than continuous normally distributed measurements, we did not remove participants through conventional z-score or Mahalanobis-distance rules. An unusual but permissible profile may represent a young person’s genuine experience rather than an error. Following Aguinis et al. (2013), we therefore distinguished invalid observations from unusual but potentially meaningful ones and excluded no participant solely for an atypical response pattern. Robust ordinal WLSMV modelling was used to limit undue statistical influence. Across the 54 substantive items, 657 of 50,274 possible responses were missing after quality checks (1.31%). Complete item data were available for 620 participants (66.6%); the other 311 participants typically missed only one item. Item-level missingness ranged from 0.11% to 2.69%. Rates were almost identical for girls (1.31%) and boys (1.30%), although younger participants had modestly more missing responses (Spearman ρ = −0.195, *p* < 0.001). We did not replace missing responses with means or medians. Because missingness was related to age and partly arose from quality recoding, MCAR was not assumed, and the available data cannot establish MAR. Ordinal analyses used pairwise-present information to estimate the correlation matrices. The lowest response category was rarely selected for items 1, 4, 5, 8, 14, 20, 23, 33, and 45, so categories 1 and 2 were combined for these nine of the 60 administered items (15.0%). Seven of the 54 substantive items used in the structural models were affected (items 1, 4, 5, 14, 20, 23, and 45; 13.0%); items 8 and 33 belonged to the Positive Impression scale and were excluded from those models. The other 47 substantive items retained four categories. This single rule was applied in both subsamples and every age and sex group. We did not fit separate four-category sensitivity models because the sparse or empty cells would not support stable threshold estimation. Comparisons with earlier studies or other language versions must therefore remain at the level of broad structure and reliability, not exact item thresholds or response frequencies. Detailed item-level distributions, missingness, and category recoding are reported in Supplementary Table S1.

### 2.5. Statistical Analysis

#### 2.5.1. Software and Reproducibility

Analyses were performed in R version 4.6.1 (R Core Team, 2026). The source data file was prepared and stored using IBM SPSS Statistics 30 (Windows) and imported into R using haven package (version 2.5.5) (Wickham et al., 2026). SPSS was not used for the psychometric or inferential analyses; these were conducted in R. Data were processed using the tidyverse packages dplyr (version 1.2.1), tidyr (version 1.3.2), purrr (version 1.2.2), tibble (version 3.3.1), readr (version 2.2.0), and stringr (version 1.6.0; Wickham et al., 2019). Polychoric correlations, exploratory factor analyses, factor congruence, and reliability estimates were computed using psych version 2.6.5 (Revelle, 2026), with factor rotation implemented through GPArotation version 2026.6.1 (Bernaards & Jennrich, 2005). Confirmatory factor analysis, exploratory structural equation modelling, and multigroup analyses were conducted using lavaan version 0.7–2 (Rosseel, 2012; Rosseel et al., 2026), with additional structural equation modelling functions provided by semTools version 0.5–9 (Jorgensen et al., 2026). Results were exported to Excel workbooks using openxlsx version 4.2.8.1 (Schauberger & Walker, 2026). The random seed, item-scoring key, and modelling specifications were explicitly documented in the R scripts to support reproducibility.

The analyses were not formally preregistered. However, the analytical workflow, random seed, data-preparation decisions, item-scoring specifications, and model specifications were documented in the R scripts to support transparency and reproducibility. Detailed information on the analytical procedures and the complete R scripts used to reproduce the analyses are provided in Supplementary File S2. Using a prespecified random seed (20260725), we divided the sample while balancing the joint distribution of sex and four age bands. This produced an exploratory subsample (*n* = 468) and a confirmatory subsample (*n* = 463). Age bands served only to balance the split; age remained continuous elsewhere. The first half allowed the data to reveal possible patterns through parallel analysis, exploratory factor analysis, and related diagnostics. The second half provided a separate setting in which to compare the prespecified CFA models. We then fitted Target-ESEM independently in both halves and in the total sample. Agreement across the two halves offers an internal check that the pattern is not unique to one analysis, but it is not external validation because all participants came from the same recruitment and data-collection process.

#### 2.5.2. Ordinal Correlations and Exploratory Analyses

Because the four response options are ordered but cannot be assumed to be equally spaced, associations between items were estimated using polychoric correlations. When a particular combination of responses to two items was not observed, a small continuity correction of 0.5 was applied to the corresponding empty cell to support stable estimation; this correction did not change participants’ responses or replace missing data (Flora & Curran, 2004). We also examined whether the exploratory correlation matrix was positive definite and reported whenever smoothing was required. Horn’s parallel analysis was used to explore the number of factors by comparing the observed eigenvalues with those obtained from simulated data. Minimum-residual extraction with direct oblimin rotation was then used to examine both the theoretically expected five-factor structure and the solution suggested by parallel analysis. Rather than treating parallel analysis as an automatic decision rule, its results were considered alongside the theoretical model and the psychological interpretability of the factors. This was important because sparse ordinal responses and clusters of items with similar content can lead to the retention of more narrow factors than are substantively meaningful (Garrido et al., 2013).

#### 2.5.3. CFA and Target-ESEM

All latent-variable models treated the item variables as ordered and used the robust weighted least-squares mean- and variance-adjusted estimator (WLSMV), theta parameterization, standardized latent variances, and pairwise-present information for missing responses. In lavaan, WLSMV uses a diagonal weight matrix for estimation and the full weight matrix to obtain robust standard errors and a mean- and variance-adjusted test statistic (Rosseel, 2012). This estimator is preferable to normal-theory maximum likelihood for ordinal indicators with few categories (Flora & Curran, 2004; Li, 2016).

**Figure 1:**
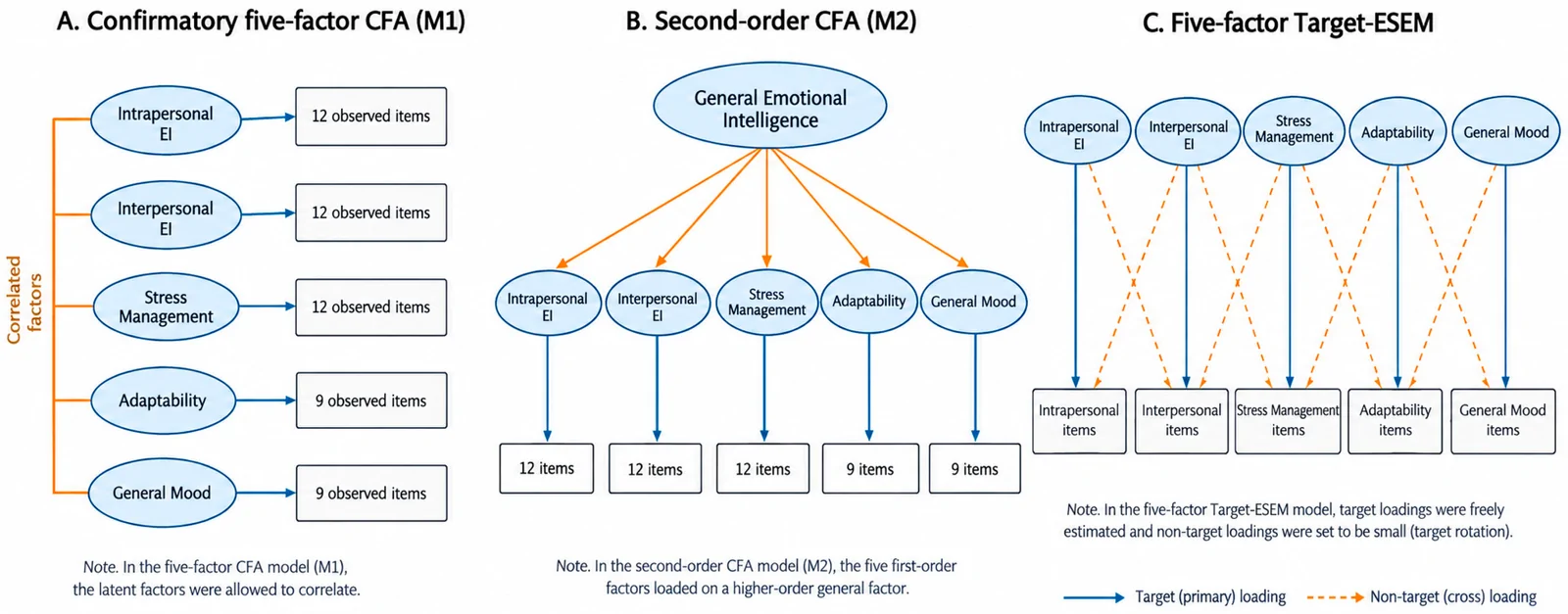
Conceptual representation of the principal factor models. Positive Impression (six items) was retained in the instrument but excluded from the substantive factor models. M3–M5 were limited item-removal sensitivity variants of M1 and are not depicted separately.

The confirmatory models were: M1, five correlated first-order factors; M2, the same five domains loading on a second-order general factor; and M3–M5, limited item-removal sensitivity models generated from local diagnostics (Figure 1). Reduced models were not regarded as candidate final forms unless improvement was substantive and the removed items showed replicated weakness outside the model in which they had been selected.

Target-ESEM was specified with five exploratory factors embedded in the structural-equation framework. For each item, the loading on its theorized domain was freely estimated (an unspecified target), whereas the four non-primary loadings were targeted toward zero. Rotation used the gradient projection algorithm, standardized observed variables, no row weighting, 100 random starts, and no post hoc reordering of latent variables. Unlike ICM-CFA, target-ESEM permits construct-relevant multidimensionality while retaining a theory-informed loading matrix (Asparouhov & Muthén, 2009; Marsh et al., 2014).

CFA and Target-ESEM offered complementary perspectives on how the items represent young people’s emotional and social functioning. CFA provided a deliberately strict test of the proposed structure by assuming that each item reflected only its intended domain, which also allowed the findings to be compared with earlier EQ-i:YV research. Target-ESEM, however, offered a more psychologically realistic representation of everyday emotional functioning because it allowed each item to relate primarily to its intended domain while also acknowledging smaller associations with other relevant competencies. For example, coping with a stressful situation may involve not only Stress Management but also emotional self-awareness, interpersonal understanding, and the flexibility needed to adjust one’s response. Target-ESEM was therefore treated as the main model for interpreting the five-domain structure, as it recognizes that emotional competencies can remain distinguishable while still operating together in children’s and adolescents’ daily lives (Marsh et al., 2014).

The analyses by sex and the sensitivity analysis excluding the youngest participants were retained as CFA-based secondary checks, allowing the findings to be examined within a conventional and more restrictive framework. These analyses were not considered direct confirmation of the preferred Target-ESEM structure. Because the baseline configural CFA did not represent the responses adequately, the comparisons between girls and boys should be interpreted as provisional rather than as evidence that measurement invariance has been established (Putnick & Bornstein, 2016). A multigroup ordinal Target-ESEM conducted with an independently recruited sample would provide a more appropriate basis for determining whether girls and boys understand and respond to the instrument in sufficiently similar ways.

Item-level stability was summarized using three descriptive flags: (a) whether the expected factor had the largest absolute loading; (b) whether the intended loading was weak (absolute loading < 0.30) or a non-target loading was salient (absolute loading ≥ 0.30); and (c) whether the two largest absolute loadings differed by less than 0.10. Loadings around 0.30 are commonly used as a minimum descriptive salience threshold in exploratory factor work, but acceptable values depend on sample size, communalities, factor complexity, and the purpose of the scale (Howard, 2016; Worthington & Whittaker, 2006). The 0.10 difference was a prespecified ambiguity flag intended to identify items whose domain assignment was not clearly separated; it was not treated as a universal psychometric cut-off. Because target-ESEM estimates the small cross-loadings expected in multidimensional constructs (Marsh et al., 2014), none of these flags was used as an automatic deletion rule. An item was prioritized for review only when the pattern replicated across the exploratory, confirmatory, and total-sample solutions and was substantively interpretable. Tucker congruence provided a complementary factor-level check; values around 0.85–0.94 were described as fair similarity and values of 0.95 or above as factor equivalence, with caution because congruence depends on factor complexity and item quality.

#### 2.5.4. Model Fit, Reliability, and Invariance

Model fit was summarized by the robust χ^2^ statistic and degrees of freedom, comparative fit index (CFI), Tucker–Lewis index (TLI), root mean square error of approximation (RMSEA) with 90% confidence interval, and standardized root mean square residual (SRMR). CFI and TLI quantify improvement over an independence model; RMSEA estimates approximate discrepancy per degree of freedom; and SRMR summarizes standardized residual discrepancies. Values near CFI/TLI ≥ 0.95, RMSEA ≤ 0.06, and SRMR ≤ 0.08 are commonly cited as reference points (Hu & Bentler, 1999), but they were not used as universal decision rules. Those cut-offs were developed under conditions that differ from large, complex ordinal models, and DWLS/WLSMV fit indices can behave differently from their maximum-likelihood counterparts (Savalei, 2021; Xia & Yang, 2019). Fit indices were therefore interpreted jointly with parameter estimates, theoretical coherence, and cross-sample replication.

Following Nye’s (2023) CFA reporting framework, we documented the rationale for the alternative measurement structures, data screening and missing-data handling, software and versions, estimator and parameterization, factor scaling, analytic sample sizes, convergence, robust test statistics and degrees of freedom, multiple approximate fit indices, the RMSEA confidence interval, standardized parameters, and all data-informed modifications. No correlated residuals were introduced. Because this psychometric analysis used a fixed existing dataset, no prospective power analysis determined the sample size. The prespecified split assessed internal stability only and was not described as external validation. Data-informed item-removal models were diagnostic sensitivity analyses and were not accepted solely because they improved global fit.

Reliability was estimated from polychoric correlation matrices. We retained ordinal alpha to remain comparable with earlier EQ-i:YV research and emphasized omega total because it does not assume that every item contributes equally. For the 54 substantive items together, omega hierarchical addressed a more practical question: how much reliable total-score variance could genuinely be attributed to one common factor after the five domains were considered (Dunn et al., 2014; Gadermann et al., 2012; Reise et al., 2013). These coefficients describe scores in this sample; they are not permanent qualities carried by the instrument into every population and use.

Convergent and discriminant validity were examined as complementary diagnostics. Average variance extracted (AVE) was computed for each domain from the squared standardized loadings of the ordinal five-factor CFA; values of 0.50 or higher indicate that a factor explains at least half of the variance in its indicators (Fornell & Larcker, 1981). Discriminant validity was evaluated using the heterotrait-monotrait ratio of correlations (HTMT), calculated from the pairwise polychoric item correlations. HTMT values below the conservative 0.85 reference value were taken as evidence that two domains were empirically distinguishable (Henseler et al., 2015). Given the ordinal items and the cross-loading patterns observed in the target-ESEM analyses, AVE and HTMT were interpreted together with the factor loadings and model-fit results rather than as stand-alone pass/fail tests.

Ordinal multi-group CFA examined configural structure, equality of thresholds, equality of thresholds plus factor loadings (labelled scalar in the output), and equality of thresholds, loadings, and residuals (strict). Changes in fit were interpreted using Chen’s (2007) commonly used sensitivity guidelines, adapted descriptively to this ordinal sequence. For threshold invariance relative to the configural model, invariance was considered tenable when the decrease in CFI did not exceed 0.010 and increases in RMSEA and SRMR did not exceed 0.015 and 0.010, respectively. When equality constraints on factor loadings were added, the corresponding limits were 0.010 for the CFI decrease, 0.015 for the RMSEA increase, and 0.030 for the SRMR increase. For strict invariance, after residual equality constraints were added, the limits were again 0.010, 0.015, and 0.010 for CFI, RMSEA, and SRMR, respectively. These values were treated as sensitivity guidelines rather than mechanical decision rules. Invariance comparisons were considered substantively interpretable only when the baseline configural representation was tenable (Putnick & Bornstein, 2016); because configural CFI and TLI were low here, the sex comparisons were retained as provisional diagnostics rather than evidence that invariance had been established. Detailed item-level and sensitivity results are reported in Supplementary Table S8.

## 3. Results

### 3.1. Exploratory Diagnostics

The exploratory correlation matrix required smoothing because it was not positive definite, most likely because some combinations of responses were rare across a large set of ordinal items. Parallel analysis suggested 11 factors. We treated this as a warning that the item set contains narrower clusters of shared content, not as a literal claim that young people’s emotional-social functioning should be divided into 11 interpretable traits. This cautious interpretation was appropriate because the correlation matrix required smoothing and item-level parallel analysis may sometimes suggest more narrowly defined factors than are substantively meaningful. In the exploratory-half Target-ESEM, 42 of the 54 items still related most strongly to their intended domain.

### 3.2. Confirmatory and Target-ESEM Models

The strict five-factor CFA (M1) captured some features of the responses—RMSEA was near 0.05—but CFI and TLI remained below conventional descriptive targets, and SRMR was near 0.09. Adding a second-order factor (M2) did not help. Removing locally problematic items produced only small improvements and did not solve the broader mismatch between the strict model and the way young people responded. In contrast, the five-factor Target-ESEM represented the data substantially better in both halves and the total sample (Table 2). The similarity of the estimates across the exploratory and confirmatory halves is more reassuring than a pattern obtained only after fitting the entire dataset.

All reported CFA and target-ESEM solutions converged. Table 2 presents the robust chi-square statistic, degrees of freedom, CFI, TLI, RMSEA with its 90% confidence interval, and SRMR for each principal model. Standardized target-ESEM loadings, latent factor correlations, and the complete CFA sensitivity comparison are reported in Supplementary Tables S2, S5, and S7, respectively. In the total-sample target-ESEM, standardized loadings ranged from −0.309 to 0.959, and latent factor correlations ranged from −0.003 to 0.367; no out-of-range standardized loadings or factor correlations were observed. Because the reduced CFA models produced only small, data-informed improvements, they were retained as sensitivity analyses rather than proposed as revised scoring models. The standardized target-ESEM factor correlations were mostly small to moderate and positive. The largest associations linked Interpersonal with Adaptability (r = 0.367), Intrapersonal with Adaptability and General Mood (both r = 0.365), and Interpersonal with General Mood (r = 0.361). Stress Management was only weakly related to Intrapersonal (r = 0.076) and was essentially unrelated to Adaptability (r = −0.003). Psychologically, this pattern suggests that the domains work together without being interchangeable: feeling effective in relationships and adapting flexibly tended to co-occur, whereas tolerating pressure or controlling impulses did not necessarily imply flexible problem-solving. The full standardized correlation table is provided in Supplementary Table S5.

### 3.3. Replication and Item-Level Findings

The broad domain pattern reproduced reasonably well across the two halves (Table 3). Tucker congruence was 0.85 for Intrapersonal, 0.95 for Interpersonal, 0.96 for Stress Management, 0.86 for Adaptability, and 0.94 for General Mood; agreement between each half and the total-sample solution ranged from 0.95 to 0.99. At item level, the intended domain carried the strongest loading for 42 of 54 items (77.8%) in the exploratory half, 47 of 54 (87.0%) in the confirmatory half, and 45 of 54 (83.3%) in the total sample. Thus, most items behaved as intended, while a meaningful minority reflected the overlap among competencies or warrant closer review. Detailed congruence coefficients across the exploratory, confirmatory, and total-sample solutions are provided in Supplementary Table S4.

Five items (3, 11, 23, 37, and 39) showed the most consistent combination of a weak intended loading and a stronger association with another domain and were classified as replicated problems. A further six items (1, 13, 15, 16, 41, and 57) showed predominantly weak-loading patterns, while ten items (2, 10, 17, 19, 20, 28, 29, 31, 50, and 53) were classified primarily by repeated cross-loading patterns. Item 32 showed a narrower pattern of ambiguity rather than a replicated problem. Its intended General Mood loading ranged from 0.249 to 0.354 across the exploratory, confirmatory, and total-sample solutions. In the total-sample target-ESEM, General Mood remained the strongest loading (0.341), but the Interpersonal cross-loading was 0.289; the 0.052 difference met the prespecified ambiguity criterion of less than 0.10. However, item 32 was not classified as having a replicated weak primary loading, a replicated cross-loading of at least 0.30, or a replicated loading on the wrong domain (Supplementary Tables S2 and S3). These patterns identify priorities for qualitative review and future independent replication; they do not, by themselves, justify deleting items from the established instrument. Notably, item 53 was not a replicated target-ESEM problem even though it appeared in one reduced CFA sensitivity model.

### 3.4. Reliability and General-Factor Evidence

The five domain scores showed adequate to strong ordinal reliability (Table 4). Across all 54 items, omega total was high (ωT = 0.939), which means that the overall composite contained substantial reliable variance. Omega hierarchical was more modest (ωH = 0.592; asymptotic estimate = 0.630): only a moderate part of that reliability could be traced to one common factor after the domains were taken into account. The attempted second-order ESEM was also unstable. Together, these findings describe a profile with a broad shared thread, not one homogeneous emotional-intelligence trait. Domain scores should therefore lead interpretation; a global score, if retained, should be described only as a secondary multidimensional summary. Complete domain and global reliability estimates are reported in Supplementary Table S6.

The second-order ESEM did not return complete global fit indices, and only Intrapersonal and Stress Management showed clear positive relations with the proposed higher-order factor. In psychological terms, the five areas could not be reduced reliably to one common dimension. This analysis therefore offers no stable basis for treating a single total as the primary description of a young person’s emotional-social functioning.

The domains were related without becoming statistically indistinguishable. HTMT ranged from 0.311 for Stress Management–Adaptability to 0.660 for Adaptability–General Mood, and every pair remained below 0.85. AVE, however, was below 0.50 for each domain: Intrapersonal = 0.396, Interpersonal = 0.384, Stress Management = 0.239, Adaptability = 0.346, and General Mood = 0.377. The items therefore shared only modest variance with their intended domain, especially for Stress Management, even though the five domains remained distinguishable from one another. These two findings should be read together rather than as contradictory pass/fail verdicts (Supplementary Table S12).

### 3.5. Measurement Invariance by Sex

Before comparing equality constraints across sex, we examined the strict five-factor CFA separately for girls and boys. Fit was somewhat stronger for girls (*n* = 495), robust χ^2^(1367) = 3087.67, CFI = 0.818, TLI = 0.809, RMSEA = 0.050, and SRMR = 0.088, than for boys (*n* = 436), robust χ^2^(1367) = 3383.53, CFI = 0.779, TLI = 0.768, RMSEA = 0.058, and SRMR = 0.092. The unconstrained multigroup model produced CFI = 0.799, TLI = 0.789, RMSEA = 0.054, and SRMR = 0.089. Adding threshold, loading, and residual constraints did not worsen relative fit in the usual way; CFI even increased slightly. Nevertheless, the baseline model already represented responses imperfectly, especially for boys (Table 5). The absence of further deterioration cannot by itself show that girls and boys understand every item in the same way. These comparisons are therefore provisional, and a multigroup Target-ESEM or alignment analysis in a new confirmatory sample would provide a more appropriate test. The complete sequence of provisional CFA comparisons by sex is provided in Supplementary Table S8.

### 3.6. Age Sensitivity

We also asked whether the youngest eligible participants were responsible for the weak CFA fit. Repeating the analysis without all 112 eight-year-olds barely changed the results: CFI moved from 0.783 to 0.785, RMSEA stayed at 0.059, and SRMR changed from 0.083 to 0.084. Excluding these children would therefore have narrowed the developmental coverage without improving the model. Their responses were retained, while the need to examine how younger children understand particular items remains an important question for future cognitive interviewing. Full results of this age-sensitivity analysis are reported in Supplementary Table S9.

## 4. Discussion

### 4.1. Structural Validity of the Five-Domain Model

The central result is easier to understand as a statement about young people’s emotional lives than as a contest between statistical models. Responses did support the five intended domains, but they did not fall into five sealed psychological compartments. A young person who describes coping flexibly may also draw on mood, interpersonal confidence, or awareness of internal experience. Target-ESEM represented this realistic overlap by estimating small cross-loadings, whereas the more restrictive CFA required every item to reflect only one domain (Marsh et al., 2014). The better target-ESEM fit therefore supports the five-domain organization while also showing that emotional-social competencies are interconnected.

This interpretation is consistent with earlier EQ-i:YV research while also extending it. Previous exploratory studies identified five components in Portuguese and Spanish samples, broadly corresponding to the Intrapersonal, Interpersonal, Stress Management, Adaptability, and General Mood domains (Candeias et al., 2011; Ferrándiz et al., 2012; Sáinz et al., 2014). Because these exploratory approaches allowed items to relate to more than one domain, they were better able to capture the natural overlap among emotional-social competencies. By contrast, the more restrictive ICM-CFA constrained all non-target loadings to zero. The better fit obtained with Target-ESEM therefore helps explain why the five-domain structure found support in earlier exploratory studies but was not adequately represented by the restrictive CFA model in the Portuguese sample. The present study adds to this evidence by treating the four-category item responses as ordinal and assessing the replication of the structure across two independently stratified subsamples. Supplementary Table S11 summarizes the principal studies used for this comparative interpretation.

The findings should not be compared uncritically with studies of the short form. Navarro-Roldán et al. (2023) obtained a well-fitting four-factor, 21-item Colombian solution, and Henning et al. (2026) supported a 30-item four-factor Canadian version with invariance across gender and education level. Those instruments omit General Mood as a substantive domain and contain substantially fewer indicators, which reduces model complexity and the opportunity for accumulated local misfit. Their results demonstrate the viability of concise four-domain scores; they do not show that the 60-item full form should be forced into the same structure.

### 4.2. Factor Replicability and Item-Level Findings

Overall, the factor pattern provided encouraging support for the proposed structure, although it also showed that young people’s emotional and social experiences cannot always be divided into completely separate domains. In the total-sample solution, 45 of the 54 items (83.3%) were most strongly associated with their intended domain, whereas nine items (16.7%) showed a stronger relationship with another domain. Rather than indicating automatically that these items function poorly, this pattern may reflect the way emotional competencies come together in everyday situations; for example, adapting to a difficult event may also require managing stress, understanding one’s own emotional response, and considering the reactions of other people (Marsh et al., 2014).

The domain scores should therefore be understood as related perspectives on emotional-social functioning rather than as pure and isolated psychological traits. When interpreting a young person’s profile, an unexpected item response may be more meaningful when considered alongside the pattern across conceptually related domains, the specific content of the item, the young person’s developmental level, and the family and school contexts in which their emotional experiences occur. This contextual and developmentally sensitive approach is also consistent with contemporary standards for responsible psychological assessment (American Educational Research Association et al., 2014).

Five indicators—items 3, 11, 23, 37, and 39—showed the clearest replicated combination of weak intended loadings and stronger associations with another domain. Item 41 showed a predominantly weak-loading pattern but did not meet the stricter replicated-problem classification. The convergence with Ferrándiz et al. (2012) is especially informative: items 3 and 39 also had unusually weak item-total relations in their Spanish validation. This replication across neighbouring linguistic and cultural contexts strengthens the case for reviewing the translation, developmental accessibility, and content alignment of those two indicators. Items 19, 20, and 29 showed replicable cross-loadings, which may reflect genuine overlap between mood, interpersonal functioning, and adaptive coping rather than defective wording.

At the same time, the evidence argues against immediate deletion. First, reduced CFA models improved CFI/TLI only modestly and did not produce clearly good fit. Second, item 53 appeared problematic in one post hoc CFA model but not in the replicated target-ESEM, illustrating how deletion decisions can depend on the selected model and sample. Third, removing established items changes content coverage and breaks comparability with international norms. A defensible Portuguese short form would require a new developmental process: qualitative item review, cognitive interviewing across age groups, independent calibration, and validation against external criteria. The current findings provide a prioritized item-review list, not a revised test.

Item 32 illustrates why a graded, replication-based decision rule is preferable to deletion based on a single loading pattern. Its General Mood loading was the largest in the total sample, and the observed ambiguity arose because a moderate Interpersonal loading was close to, but did not exceed, the intended loading. This may reflect substantive overlap between positive mood and perceived interpersonal functioning rather than a poorly functioning item. Moreover, the primary loading varied around the 0.30 descriptive threshold across the split samples, and none of the stronger problem criteria replicated. Deleting the item on this evidence would risk capitalizing on small sample-specific fluctuations, narrowing General Mood content, and reducing comparability with the established full form. We therefore retained item 32 while identifying it for cognitive interviewing and independent cross-validation, particularly across age groups.

### 4.3. Reliability, General Factor, and Score Interpretation

Domain reliability compared favorably with previous work. The Portuguese ordinal alpha/omega estimates ranged from approximately 0.74/0.76 for Intrapersonal to 0.87/0.87 for Interpersonal. Ferrándiz et al. (2012) reported alpha values from 0.63 to 0.80 for the Spanish full form, while the Colombian short-form omega estimates ranged from 0.57 to 0.75 (Navarro-Roldán et al., 2023). Some advantage in the present estimates is expected because ordinal coefficients use polychoric rather than Pearson correlations and can be higher when categories are few or skewed (Gadermann et al., 2012). Reliability comparisons must therefore consider the item set, coefficient, correlation metric, and sample.

For practice, the evidence supports a clear hierarchy of interpretation. The five domain scores should be primary because they preserve meaningful differences in how a young person understands emotions, relationships, pressure, problem-solving, and general mood. The high omega total shows that the 54-item composite is reliable in a broad sense, but omega hierarchical of 0.592 indicates that only a moderate share of reliable total-score variance can be attributed to one general factor. Together with the unstable higher-order ESEM, this does not support using the global score as a stand-alone indicator of emotional intelligence (Reise et al., 2013; Rodriguez et al., 2016). If continuity with previous reports requires a global score, it should be labelled as a broad descriptive composite, presented alongside the domain profile, and never used alone for diagnosis, eligibility, or individual intervention decisions.

The correlation pattern further explains why the profile matters. Interpersonal, Adaptability, General Mood, and Intrapersonal scores shared modest positive associations, suggesting related but distinguishable ways of experiencing emotional-social competence. Stress Management was much less connected to the other domains and was virtually unrelated to Adaptability. A child may therefore tolerate frustration or control impulses without necessarily feeling flexible in solving problems, just as another may be resourceful in new situations but still struggle under emotional pressure. The cross-loadings tell a complementary story: some responses appear to draw on more than one capacity. Such overlap is psychologically plausible and should prompt contextual interpretation rather than automatic item deletion (Marsh et al., 2014).

The construct-validity diagnostics refine this interpretation. HTMT values below 0.85 indicate that the five domains remain empirically distinguishable despite their conceptual relatedness. At the same time, AVE values from 0.239 to 0.396 show that no domain explained half of the variance in its designated items. This is not a contradiction: the domains can be distinguishable from one another while their indicators still show modest convergence on the intended factor. The low AVE estimates are consistent with the modest primary loadings and meaningful cross-loadings identified by target-ESEM and are most pronounced for Stress Management. They strengthen the case for domain-level interpretation with caution, item-level qualitative review, and independent replication rather than immediate item deletion.

### 4.4. Measurement Invariance and Developmental Considerations

Equality constraints across sex did not worsen the ordinal CFA indices, which is compatible with—though weaker than—the invariance evidence reported for the recent Canadian short form (Henning et al., 2026). The limitation is that the Portuguese configural CFA already had weak CFI/TLI. Invariance is not established merely because a constrained model fails to deteriorate when the baseline representation is misspecified. The present results should therefore be described as provisional sensitivity evidence, not as proof that all item thresholds and loadings are equivalent for girls and boys. A multi-group target-ESEM in an independent sample is the logical next test.

Valid eight-year-old participants were retained. Excluding them did not improve CFA fit, so a statistical age cut-off would have sacrificed coverage without solving the structural problem. This is compatible with the intended 7–18-year age range of the EQ-i:YV. Nevertheless, age appropriateness cannot be inferred from model fit alone. Younger respondents may understand particular abstract or negatively formulated statements differently; future cognitive interviews should examine response processes directly. The heterogeneous developmental patterns found by Esnaola et al. (2017) also argue against assuming a simple linear maturation of every perceived EI domain.

### 4.5. Practical Implications for Psychological and Educational Assessment

Portuguese psychologists should therefore organize feedback around the five-domain profile. Rather than reducing a young person’s functioning to one rank or label, the profile can identify relative strengths and areas in which support may be useful. These scores describe self-perceived competencies; they are not diagnoses or direct demonstrations of emotional performance. Interpretation should be integrated with the referral question, developmental history, interview material, and the contexts in which the young person lives and learns, in line with contemporary standards for psychological testing (American Educational Research Association et al., 2014).

Educational and clinical psychologists do not need to erase previously reported total scores, but they should change the weight given to them. A total score may remain in a report for descriptive continuity only if its multidimensional meaning is stated and the domain pattern is shown. It should not determine placement, diagnosis, access to support, or treatment planning by itself. Because children may experience and express difficulties differently at home, at school, and with peers, self-report should also be considered together with interviews and, when appropriate, information from parents, teachers, or other observers (De Los Reyes et al., 2015).

**Figure 2:**
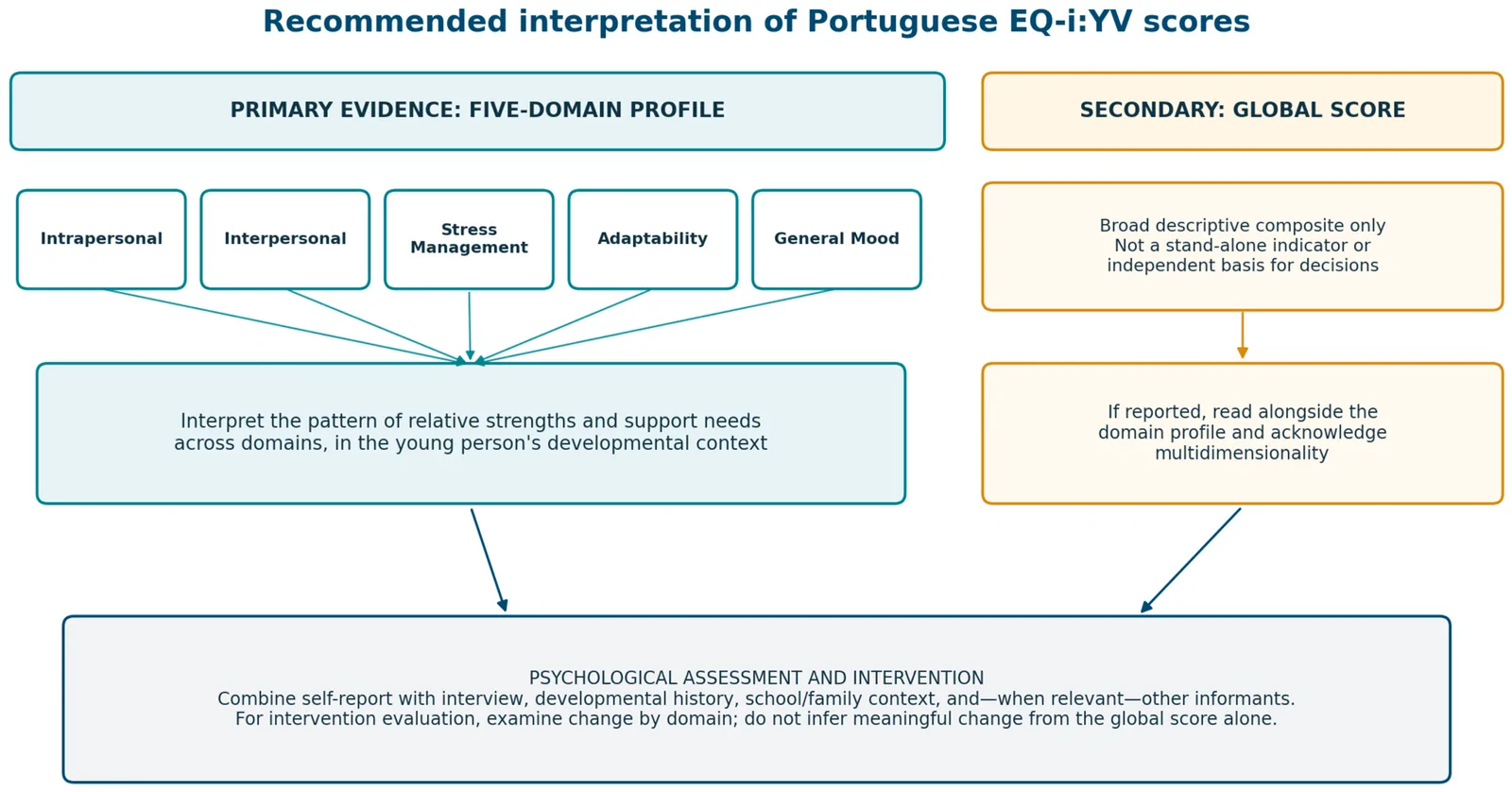
Recommended hierarchy for interpreting Portuguese EQ-i:YV scores in psychological and educational assessment.

For intervention evaluation, change should be examined domain by domain. An intervention may strengthen stress tolerance without changing interpersonal confidence, or improve problem-solving while mood remains unchanged; a single total score could hide these clinically and educationally meaningful patterns. Until test–retest reliability, measurement invariance, and sensitivity to change have been established in Portuguese samples, score differences over time should be interpreted cautiously and combined with other evidence of change (American Educational Research Association et al., 2014). Figure 2 summarizes this recommended interpretive hierarchy.

### 4.6. Limitations and Future Research

Several limitations mark where interpretation should stop. Recruitment depended on schools’ collaboration, so findings cannot automatically be extended to every Portuguese child or adolescent. Public, private, and cooperative schools from three regions were included, but the dataset did not retain socioeconomic, ethnic, migration, or school-deprivation indicators. These results are not national norms. Dividing one sample into two halves showed internal stability within this recruitment process; it did not replace validation in a newly recruited, more representative group.

The study also heard only the young person’s perspective. That perspective is central to the EQ-i:YV, but self-description changes with insight, language development, response style, and the setting in which questions are answered. Without reports from parents or teachers, observation, performance tasks, or external criteria, we could not examine where perspectives converge or test criterion-related, predictive, and incremental validity (De Los Reyes et al., 2015). Positive Impression was excluded from the factor models because it serves mainly as a response-style indicator, but its practical value in Portuguese assessment also remains to be studied.

Finally, one assessment occasion cannot tell us whether scores remain stable, respond meaningfully to intervention, or follow a consistent developmental course. Sparse response categories and the fixed collapsing rule may have influenced polychoric estimates and limit direct threshold comparisons with other studies. The CFA-based sex and age analyses were secondary checks and did not fully match the better-fitting Target-ESEM. Future studies should prioritize multigroup ordinal Target-ESEM, test–retest reliability, item response theory, differential item functioning, cognitive interviews, and external convergent and criterion evidence. Missingness was low (1.31%), but its association with age means that MCAR cannot be assumed and MAR cannot be verified.

## 5. Conclusions

The Portuguese EQ-i:YV is most useful as a profile of five related ways in which young people see their emotional and social functioning. Target-ESEM described that profile more convincingly than the restrictive CFA because it allowed the modest overlap expected in real emotional life. The five domain scores were adequately to strongly reliable and should guide interpretation. By contrast, the moderate omega hierarchical and unstable higher-order model do not support reducing a young person’s responses to one stand-alone trait score. If a global score is reported, it should remain a secondary broad description placed beside the domain pattern. The full form can continue to support research, provided that interpretation stays psychologically informed, developmentally sensitive, and grounded in context rather than becoming a mechanical label. Independent replication, external validity evidence, longitudinal reliability, and multigroup Target-ESEM are still needed before stronger clinical or educational uses can be justified (American Educational Research Association et al., 2014; Reise et al., 2013).

## Figures and Tables

**Table 1:** Sample distribution by age group and sex.

Age Group (Years)	Girls, *n*	Boys, *n*	Total, *n*	% of Sample
8–10	179	155	334	35.9
11–12	114	106	220	23.6
13–14	147	104	251	27.0
15–16	55	71	126	13.5
Total	495	436	931	100.0

**Table 2:** Fit of confirmatory and target-ESEM models.

Model/Sample	*n*	χ^2^	df	CFI	TLI	RMSEA [90% CI]	SRMR
CFA M1: 5 correlated factors/CFA half	463	3184.263	1367	0.802	0.793	0.054 [0.051, 0.056]	0.089
CFA M2: second order/CFA half	463	3200.441	1372	0.801	0.793	0.054 [0.051, 0.056]	0.090
CFA M4b: remove 15, 37, 53/CFA half	463	2854.367	1214	0.819	0.809	0.054 [0.052, 0.057]	0.088
Target-ESEM: 5 factors/EFA half	468	1933.382	1171	0.921	0.903	0.037 [0.034, 0.040]	0.053
Target-ESEM: 5 factors/CFA half	463	1977.785	1171	0.912	0.893	0.039 [0.036, 0.042]	0.054
Target-ESEM: 5 factors/total sample	931	2878.606	1171	0.917	0.898	0.040 [0.038, 0.041]	0.045

Note: CFA = confirmatory factor analysis; ESEM = exploratory structural equation modelling; CFI = comparative fit index; TLI = Tucker–Lewis index; RMSEA = root mean square error of approximation; SRMR = standardized root mean square residual. M4b is reported only as a sensitivity model and is not proposed as a revised scoring form.

**Table 3:** Replicability of the target-ESEM factors.

Factor	EFA–CFA Congruence	EFA–Total	CFA–Total
Intrapersonal	0.85	0.95	0.97
Interpersonal	0.95	0.99	0.98
Stress Management	0.96	0.99	0.99
Adaptability	0.86	0.97	0.95
General Mood	0.94	0.98	0.99

**Table 4:** Ordinal reliability of the five substantive domains.

Domain	Items	Ordinal α	ω Total
Intrapersonal	6	0.744	0.760
Interpersonal	12	0.865	0.866
Stress Management	12	0.764	0.773
Adaptability	10	0.831	0.834
General Mood	14	0.854	0.860

**Table 5:** Provisional multi-group CFA by sex.

Model	CFI	TLI	RMSEA	SRMR	ΔCFI	ΔRMSEA	ΔSRMR
Configural	0.799	0.789	0.0542	0.0894	—	—	—
Thresholds	0.798	0.792	0.0539	0.0894	−0.001	0.000	0.0000
Scalar	0.808	0.806	0.0520	0.0901	+0.010	−0.002	+0.001
Strict	0.818	0.819	0.0502	0.0922	+0.010	−0.002	+0.002

Note: CFI = comparative fit index; TLI = Tucker–Lewis index; RMSEA = root mean square error of approximation; SRMR = standardized root mean square residual; delta denotes change from the immediately preceding model. Sensitivity limits were: thresholds versus configural, CFI decrease no greater than 0.010, RMSEA increase no greater than 0.015, and SRMR increase no greater than 0.010; scalar versus thresholds, 0.010, 0.015, and 0.030, respectively; strict versus scalar, 0.010, 0.015, and 0.010, respectively (Chen, 2007). Because the configural model showed limited fit, these comparisons are provisional and do not establish measurement invariance.

## Data Availability

The data are not publicly available because they concern minors and are subject to ethical and privacy restrictions. De-identified analytic materials may be considered upon reasonable request and approval by the relevant data controller. The Portuguese version of the questionnaire and its items are not included in the supplementary materials and should be requested directly from Multi-Health Systems Inc. (MHS), the copyright holder and publisher of the EQ-i:YV.

## Supplementary Materials

The following supporting information can be downloaded at: https://www.mdpi.com/article/10.3390/ejihpe16090139/s1, Supplementary File S1 contains the item-level audit and ordinal descriptive statistics (Table S1), total-sample Target-ESEM loadings (Table S2), replicated item-stability flags (Table S3), factor-congruence coefficients (Table S4), standardized latent factor correlations (Table S5), reliability estimates (Table S6), CFA and item-deletion sensitivity comparisons (Table S7), provisional CFA comparisons by sex (Table S8), age-sensitivity results (Table S9), the numerical scoring key used in the structural analyses (Table S10), comparative evidence from selected EQ-i:YV studies (Table S11), and AVE and HTMT results (Table S12). Supplementary File S2 contains the R scripts for data screening, stratified sample splitting, ordinal EFA, CFA, reliability, age-sensitivity, ESEM, replication, and final Target-ESEM analyses. The scripts document the fixed random seed (20260725), data-preparation decisions, the numerical item-scoring key without copyrighted wording, and the complete modelling specifications. Participant-level data are not included because the dataset concerns minors and is subject to ethical and privacy restrictions.

## Abbreviations

The following abbreviations and statistical symbols are used in this manuscript:

CFA	Confirmatory factor analysis
CFI	Comparative fit index
CNPD	Portuguese Data Protection Commission
DWLS	Diagonally weighted least squares
EFA	Exploratory factor analysis
EI	Emotional intelligence
EQ-i:YV	Emotional Quotient Inventory: Youth Version
ESEM	Exploratory structural equation modelling
FCT	Fundação para a Ciência e a Tecnologia
ICM-CFA	Independent-clusters confirmatory factor analysis
MIME	Monitoring of Surveys in School Environments
RMSEA	Root mean square error of approximation
SEM	Structural equation modelling
SRMR	Standardized root mean square residual
TLI	Tucker–Lewis index
WLSMV	Weighted least squares mean- and variance-adjusted
α	Cronbach’s alpha
ω	Omega total for a domain score
ωH	Omega hierarchical
ωT	Omega total for the complete item set
