# Supplementary material for: Assessing Emotional and Social Intelligence in Portuguese Youth: Structural Validity of the EQ-i:YV
Source: Eur J Investig Health Psychol Educ. 2026 Sep 13;16(9):139. doi: 10.3390/ejihpe16090139 (PMC13605546; doi:10.3390/ejihpe16090139)
Supplement: Supplementary file 1 [file ejihpe-16-00139-s001.zip › ejihpe-4493429-supplementary.pdf]

European Journal of Investigation in Health, Psychology and Education

MDPI

Supplementary Materials

European Journal of Investigation in Health, Psychology and Education

Special Issue: Emotional Intelligence Development in Youth

Assessing Emotional and Social Intelligence in Portuguese Youth: Structural Validity of the EQ-i:YV

Adelinda A. Candeias et al.

This supplementary file reports the detailed item-level, structural, reliability, replication, sensitivity, and comparative results supporting the main manuscript.

Table S1. Item-level audit and ordinal descriptive statistics

Item	Valid n	M	SD	Skew	Kurt.	n(1)	n(2)	n(3)	n(4)
1	929	3.86	0.42	-3.04	8.80	0	25	83	821
2	930	3.00	0.70	-0.21	-0.36	11	193	508	218
3	923	2.79	0.88	-0.19	-0.78	65	281	360	217
4	928	3.52	0.65	-1.01	-0.11	0	79	287	562
5	924	3.35	0.73	-0.66	-0.88	0	142	314	468
6	918	2.77	0.94	-0.41	-0.69	111	201	396	210
7	919	2.45	0.89	0.22	-0.71	119	403	263	134
8	926	3.55	0.67	-1.18	0.10	0	96	227	603
9	918	3.15	0.83	-0.54	-0.67	23	189	336	370
10	918	2.91	0.76	-0.13	-0.63	19	253	442	204
11	920	2.98	0.85	-0.31	-0.84	33	245	352	290
12	919	2.91	0.82	-0.16	-0.83	28	270	378	243
13	926	2.71	0.76	0.06	-0.57	32	343	411	140
14	915	3.35	0.72	-0.63	-0.86	0	132	332	451
15	918	2.37	0.81	-0.22	-0.70	150	325	399	44
16	920	2.90	0.81	-0.22	-0.69	33	256	400	231
17	915	2.37	0.89	0.24	-0.65	144	401	261	109
18	922	2.89	0.76	-0.27	-0.34	31	230	467	194
19	925	3.05	0.81	-0.38	-0.68	25	211	386	303
20	920	3.85	0.45	-3.15	9.09	0	35	64	821
21	906	2.99	0.83	-0.71	0.18	62	127	471	246
22	922	2.58	0.76	0.31	-0.51	38	427	341	116
23	919	3.79	0.52	-2.40	4.77	0	46	103	770
24	920	3.32	0.86	-1.02	0.02	34	137	247	502
25	909	3.15	0.82	-0.51	-0.72	19	188	338	364

Item	Valid n	M	SD	Skew	Kurt.	n(1)	n(2)	n(3)	n(4)
26	919	2.94	0.88	-0.69	-0.08	84	133	459	243
27	919	2.26	0.74	0.55	0.23	103	540	214	62
28	919	2.52	0.91	-0.22	-0.80	153	249	401	116
29	922	2.99	0.83	-0.27	-0.88	25	246	362	289
30	920	2.70	0.76	0.22	-0.70	23	376	377	144
31	924	2.35	0.87	0.33	-0.53	137	437	244	106
32	929	3.39	0.73	-0.96	0.18	12	103	322	492
33	926	3.61	0.62	-1.35	0.66	0	70	222	634
34	920	2.87	0.83	-0.09	-0.89	29	295	365	231
35	915	2.79	0.88	-0.53	-0.33	100	172	464	179
36	911	3.37	0.77	-1.04	0.36	20	105	300	486
37	910	3.18	1.05	-1.05	-0.20	120	71	244	475
38	917	2.67	0.79	0.24	-0.76	32	395	334	156
39	912	2.54	0.95	0.15	-0.95	115	374	242	181
40	919	3.20	0.84	-0.69	-0.48	26	168	317	408
41	913	3.17	0.83	-0.59	-0.62	23	180	327	383
42	918	1.77	0.87	1.01	0.30	424	337	101	56
43	914	2.29	0.89	0.43	-0.51	157	446	198	113
44	921	2.77	0.83	0.09	-0.92	32	351	337	201
45	917	3.32	0.79	-0.64	-1.11	0	185	249	483
46	915	2.90	0.93	-0.53	-0.57	90	178	383	264
47	915	3.43	0.83	-1.23	0.39	24	128	190	573
48	907	2.62	0.80	0.30	-0.69	39	415	309	144
49	911	2.71	0.91	-0.39	-0.61	113	208	419	171
50	918	3.05	0.85	-0.77	0.14	65	118	445	290
51	918	3.61	0.69	-1.72	2.19	12	75	172	659
52	910	2.48	0.86	0.38	-0.62	82	455	230	143
53	918	2.59	0.88	-0.39	-0.59	136	217	456	109
54	919	2.86	0.87	-0.60	-0.19	89	157	471	202
55	922	3.27	0.80	-0.74	-0.35	18	148	327	429
56	920	3.05	0.98	-0.57	-0.87	68	212	248	392
57	916	2.95	0.95	-0.43	-0.86	70	227	302	317
58	924	2.67	0.97	-0.29	-0.88	137	223	371	193

Item	Valid n	M	SD	Skew	Kurt.	n(1)	n(2)	n(3)	n(4)
59	923	3.00	0.86	-0.35	-0.86	33	240	340	310
60	928	3.14	0.92	-0.62	-0.79	41	211	254	422

Note: Item wording is intentionally omitted. Statistics describe the final analytic data. Categories 1 and 2 were combined for nine of the 60 administered items (items 1, 4, 5, 8, 14, 20, 23, 33, and 45). Seven of these were among the 54 substantive items modelled (items 1, 4, 5, 14, 20, 23, and 45); items 8 and 33 belonged to Positive Impression and were excluded from the structural models. Missing responses were excluded from item-level calculations.

Table S2. Target-ESEM standardized loadings in the total sample

Item	Expected domain	Intrapersonal	Interpersonal	Stress Management	Adaptability	General Mood	Strongest loading	Expected domain strongest?
7	Intrapersonal	0.595	0.078	-0.043	0.032	0.065	Intrapersonal	Yes
17	Intrapersonal	0.689	0.120	-0.074	0.006	0.011	Intrapersonal	Yes
28	Intrapersonal	0.418	-0.025	0.237	-0.309	0.050	Intrapersonal	Yes
31	Intrapersonal	0.669	-0.012	-0.055	0.036	0.085	Intrapersonal	Yes
43	Intrapersonal	0.720	0.023	-0.088	0.017	0.038	Intrapersonal	Yes
53	Intrapersonal	0.380	-0.075	0.262	-0.251	0.101	Intrapersonal	Yes
2	Interpersonal	0.228	0.516	-0.051	0.144	-0.118	Interpersonal	Yes
5	Interpersonal	0.112	0.645	-0.027	0.061	-0.077	Interpersonal	Yes
10	Interpersonal	0.244	0.533	-0.034	0.203	-0.176	Interpersonal	Yes
14	Interpersonal	0.035	0.517	0.172	0.107	0.006	Interpersonal	Yes
20	Interpersonal	-0.153	0.463	0.153	-0.068	0.303	Interpersonal	Yes
24	Interpersonal	-0.035	0.503	0.145	0.050	-0.014	Interpersonal	Yes
36	Interpersonal	0.048	0.681	0.043	-0.048	-0.002	Interpersonal	Yes
41	Interpersonal	0.147	0.238	0.036	0.081	0.341	General Mood	No
45	Interpersonal	-0.050	0.573	0.084	0.003	0.067	Interpersonal	Yes
51	Interpersonal	-0.112	0.636	0.034	-0.139	0.246	Interpersonal	Yes
55	Interpersonal	-0.021	0.635	-0.048	0.083	0.055	Interpersonal	Yes
59	Interpersonal	0.005	0.501	-0.058	0.236	0.037	Interpersonal	Yes
3	Stress Management	0.017	0.214	0.094	0.241	-0.016	Adaptability	No
6	Stress Management	-0.071	0.028	0.476	0.097	-0.078	Stress Management	Yes
11	Stress Management	0.082	0.141	0.198	0.320	0.152	Adaptability	No
15	Stress Management	0.024	-0.052	0.227	-0.089	0.058	Stress Management	Yes

Item	Expected domain	Intrapersonal	Interpersonal	Stress Management	Adaptability	General Mood	Strongest loading	Expected domain strongest?
21	Stress Management	-0.083	0.171	0.565	-0.013	-0.107	Stress Management	Yes
26	Stress Management	-0.036	0.084	0.632	0.012	0.023	Stress Management	Yes
35	Stress Management	0.058	-0.030	0.705	-0.034	0.009	Stress Management	Yes
39	Stress Management	0.078	-0.033	0.135	0.258	0.074	Adaptability	No
46	Stress Management	0.027	0.042	0.549	-0.066	-0.017	Stress Management	Yes
49	Stress Management	-0.023	0.024	0.509	-0.006	-0.076	Stress Management	Yes
54	Stress Management	0.154	-0.025	0.672	-0.032	0.070	Stress Management	Yes
58	Stress Management	-0.068	-0.037	0.555	-0.030	0.057	Stress Management	Yes
12	Adaptability	0.010	0.192	0.028	0.500	0.010	Adaptability	Yes
16	Adaptability	0.143	0.047	-0.017	0.183	0.240	General Mood	No
22	Adaptability	0.037	-0.021	0.030	0.624	0.033	Adaptability	Yes
25	Adaptability	-0.030	0.248	0.040	0.471	0.014	Adaptability	Yes
30	Adaptability	0.023	-0.071	0.019	0.675	0.098	Adaptability	Yes
34	Adaptability	0.001	0.111	0.039	0.505	0.079	Adaptability	Yes
38	Adaptability	0.017	0.133	-0.069	0.443	0.073	Adaptability	Yes
44	Adaptability	0.004	0.166	-0.003	0.498	0.101	Adaptability	Yes
48	Adaptability	0.004	-0.023	-0.003	0.567	0.174	Adaptability	Yes
57	Adaptability	-0.061	0.181	-0.011	0.240	0.199	Adaptability	Yes
1	General Mood	0.061	0.278	0.024	0.039	0.228	Interpersonal	No
4	General Mood	0.177	0.206	0.054	-0.074	0.396	General Mood	Yes
9	General Mood	0.147	0.021	-0.023	0.083	0.566	General Mood	Yes
13	General Mood	0.138	0.000	-0.091	0.239	0.209	Adaptability	No
19	General Mood	0.127	-0.166	0.179	0.378	0.428	General Mood	Yes
23	General Mood	-0.035	0.490	-0.041	-0.070	0.159	Interpersonal	No
29	General Mood	0.134	-0.190	0.243	0.393	0.452	General Mood	Yes
32	General Mood	0.028	0.289	0.042	0.135	0.341	General Mood	Yes

Item	Expected domain	In-trapersonal	Interper-sonal	Stress Manage-ment	Adapta-bility	General Mood	Strongest load-ing	Expected do-main strongest?
37	General Mood	0.072	0.090	0.413	-0.081	0.072	Stress Manage-ment	No
40	General Mood	0.095	0.076	-0.014	0.067	0.630	General Mood	Yes
47	General Mood	-0.036	0.126	-0.007	-0.109	0.736	General Mood	Yes
50	General Mood	-0.126	-0.020	0.270	-0.060	0.389	General Mood	Yes
56	General Mood	-0.034	-0.080	-0.174	-0.109	0.919	General Mood	Yes
60	General Mood	-0.026	-0.098	-0.163	-0.027	0.959	General Mood	Yes

Note: The theoretical primary loading was freely estimated, and all cross-loadings were targeted toward zero. Absolute values were used to identify the strongest factor. Signs should be interpreted in conjunction with the finalized scoring key.

Table S3. Replicated item-stability flags across exploratory, confirmatory, and total samples

Item	Expected domain	Replicated classifica-tion	Minimum pri-mary loading	Maximum primary loading	Repli-cated problem	Repli-cated weak loading	Replicated cross-load-ing
37	General Mood	Replicated problem	0.047	0.112	Yes	Yes	Yes
3	Stress Management	Replicated problem	0.055	0.100	Yes	Yes	No
39	Stress Management	Replicated problem	0.121	0.150	Yes	Yes	No
23	General Mood	Replicated problem	0.134	0.226	Yes	Yes	Yes
11	Stress Management	Replicated problem	0.155	0.211	Yes	Yes	Yes
16	Adaptability	Replicated weak loading	0.106	0.388	No	No	No
13	General Mood	Replicated weak loading	0.151	0.284	No	Yes	No
57	Adaptability	Replicated weak loading	0.172	0.284	No	Yes	No
41	Interpersonal	Replicated weak loading	0.186	0.293	No	Yes	No
15	Stress Management	Replicated weak loading	0.207	0.267	No	Yes	No
1	General Mood	Replicated weak loading	0.224	0.295	No	Yes	No
53	Intrapersonal	Replicated cross-loading	0.013	0.409	No	No	Yes
28	Intrapersonal	Replicated cross-loading	0.100	0.436	No	No	Yes
50	General Mood	Replicated cross-loading	0.165	0.391	No	No	No
19	General Mood	Replicated cross-loading	0.208	0.553	No	No	No
29	General Mood	Replicated cross-loading	0.216	0.591	No	No	Yes
2	Interpersonal	Replicated cross-loading	0.332	0.550	No	No	Yes
10	Interpersonal	Replicated cross-loading	0.380	0.575	No	No	Yes

Item	Expected domain	Replicated classification	Minimum primary loading	Maximum primary loading	Replicated problem	Replicated weak loading	Replicated cross-loading
20	Interpersonal	Replicated cross-loading	0.421	0.510	No	No	No
31	Intrapersonal	Replicated cross-loading	0.455	0.684	No	No	No
17	Intrapersonal	Replicated cross-loading	0.469	0.734	No	No	No
32	General Mood	Replicated ambiguity	0.249	0.354	No	No	No
38	Adaptability	No replicated concern	0.310	0.572	No	No	No
7	Intrapersonal	No replicated concern	0.361	0.619	No	No	No
49	Stress Management	No replicated concern	0.363	0.590	No	No	No
4	General Mood	No replicated concern	0.380	0.503	No	No	No
6	Stress Management	No replicated concern	0.399	0.523	No	No	No
12	Adaptability	No replicated concern	0.399	0.609	No	No	No
24	Interpersonal	No replicated concern	0.399	0.591	No	No	No
44	Adaptability	No replicated concern	0.404	0.605	No	No	No
25	Adaptability	No replicated concern	0.405	0.598	No	No	No
59	Interpersonal	No replicated concern	0.448	0.555	No	No	No
47	General Mood	No replicated concern	0.451	0.793	No	No	No
45	Interpersonal	No replicated concern	0.461	0.595	No	No	No
46	Stress Management	No replicated concern	0.471	0.592	No	No	No
14	Interpersonal	No replicated concern	0.473	0.584	No	No	No
34	Adaptability	No replicated concern	0.486	0.541	No	No	No
21	Stress Management	No replicated concern	0.521	0.568	No	No	No
58	Stress Management	No replicated concern	0.527	0.555	No	No	No
43	Intrapersonal	No replicated concern	0.532	0.752	No	No	No
5	Interpersonal	No replicated concern	0.536	0.709	No	No	No
9	General Mood	No replicated concern	0.548	0.560	No	No	No
48	Adaptability	No replicated concern	0.563	0.629	No	No	No
51	Interpersonal	No replicated concern	0.567	0.628	No	No	No
22	Adaptability	No replicated concern	0.570	0.735	No	No	No
40	General Mood	No replicated concern	0.588	0.646	No	No	No
55	Interpersonal	No replicated concern	0.607	0.627	No	No	No
26	Stress Management	No replicated concern	0.618	0.640	No	No	No
36	Interpersonal	No replicated concern	0.624	0.665	No	No	No

Item	Expected domain	Replicated classification	Minimum primary loading	Maximum primary loading	Replicated problem	Replicated weak loading	Replicated cross-loading
54	Stress Management	No replicated concern	0.658	0.700	No	No	No
56	General Mood	No replicated concern	0.660	0.935	No	No	No
60	General Mood	No replicated concern	0.665	0.970	No	No	No
35	Stress Management	No replicated concern	0.680	0.728	No	No	No
30	Adaptability	No replicated concern	0.692	0.755	No	No	No

Note: The summary classification in Table S3 describes the dominant pattern across the three solutions. The final three columns apply stricter binary criteria that had to be met in every solution; consequently, a descriptive classification does not necessarily imply a 'Yes' for the corresponding strict flag.

Table S4. Target-ESEM factor congruence across samples

Factor	EFA–CFA	EFA–total	CFA–total
Intrapersonal	0.850	0.950	0.970
Interpersonal	0.950	0.990	0.980
Stress Management	0.960	0.990	0.990
Adaptability	0.860	0.970	0.950
General Mood	0.940	0.980	0.990

Table S5. Target-ESEM latent factor correlations in the total sample

Factor 1	Factor 2	r	p
Intrapersonal	Interpersonal	0.230	< 0.001
Intrapersonal	Stress Management	0.076	0.014
Intrapersonal	Adaptability	0.365	< 0.001
Intrapersonal	General Mood	0.365	< 0.001
Interpersonal	Stress Management	0.201	< 0.001
Interpersonal	Adaptability	0.367	< 0.001
Interpersonal	General Mood	0.361	< 0.001
Stress Management	Adaptability	-0.003	0.912
Stress Management	General Mood	0.164	< 0.001
Adaptability	General Mood	0.349	< 0.001

Table S6. Reliability estimates

Domain	Items	Ordinal α	ω total
Intrapersonal	6	0.744	0.760
Interpersonal	12	0.865	0.866

Domain	Items	Ordinal α	ω total
Stress Management	12	0.764	0.773
Adaptability	10	0.831	0.834
General Mood	14	0.854	0.860

Global estimates for the 54 substantive items: ω total = 0.939; ω hierarchical = 0.592; asymptotic ω hierarchical = 0.630.

Table S7. CFA model comparisons and item-deletion sensitivity

Model	χ^2	df	CFI	TLI	RMSEA	90% CI	SRMR
M1: Five correlated factors	3184.263	1367	0.802	0.793	0.054	0.051–0.056	0.089
M2: Second-order model	3200.441	1372	0.801	0.793	0.054	0.051–0.056	0.090
M3: Without items 15 and 37	3013.720	1264	0.808	0.799	0.055	0.052–0.057	0.088
M4a: Without items 15, 37, and 28	2854.932	1214	0.819	0.809	0.054	0.052–0.057	0.088
M4b: Without items 15, 37, and 53	2854.367	1214	0.819	0.809	0.054	0.052–0.057	0.088
M5: Without items 15, 37, 28, and 53	2798.905	1165	0.819	0.809	0.055	0.052–0.058	0.088

Note: Reduced models are sensitivity analyses only. Small fit changes do not constitute sufficient evidence for a revised instrument.

Table S8. Exploratory measurement-invariance results by sex

Model	CFI	TLI	RMSEA	SRMR	ΔCFI	ΔRMSEA	ΔSRMR
Configural	0.799	0.789	0.0542	0.0894	—	—	—
Thresholds	0.798	0.792	0.0539	0.0894	-0.001	0.000	0.000
Scalar	0.808	0.806	0.0520	0.0901	0.010	-0.002	0.001
Strict	0.818	0.819	0.0502	0.0922	0.010	-0.002	0.002

Note: These results are exploratory. Because the configural model showed limited overall fit, the successive equality constraints should not be interpreted as establishing measurement invariance.

Table S9. Sensitivity to excluding eight-year-old participants

Model / age range	n	CFI	TLI	RMSEA	SRMR
Original 54-item CFA / 8–16 years	931	0.783	0.773	0.059	0.083
Original 54-item CFA / 9–16 years	819	0.785	0.775	0.059	0.084
Reduced CFA without items 15, 37, and 53 / 8–16 years	931	0.800	0.790	0.059	0.082
Reduced CFA without items 15, 37, and 53 / 9–16 years	819	0.799	0.789	0.059	0.083

Note: The ten records originally coded as age 7 were verified as age 8. The 9–16 sensitivity analysis excludes all 112 valid eight-year-old participants.

Table S10. Scoring key used for the structural analyses

Scale	Item numbers	Count	Role in structural model
Intrapersonal	7, 17, 28, 31, 43, 53	6	Substantive factor

Scale	Item numbers	Count	Role in structural model
Interpersonal	2, 5, 10, 14, 20, 24, 36, 41, 45, 51, 55, 59	12	Substantive factor
Stress Management	3, 6, 11, 15, 21, 26, 35, 39, 46, 49, 54, 58	12	Substantive factor
Adaptability	12, 16, 22, 25, 30, 34, 38, 44, 48, 57	10	Substantive factor
General Mood	1, 4, 9, 13, 19, 23, 29, 32, 37, 40, 47, 50, 56, 60	14	Substantive factor
Positive Impression	8, 18, 27, 33, 42, 52	6	Retained in instrument; excluded from substantive factor models

The archived item variables were already oriented in their final scoring direction. No second reversal was applied.

Table S11. Comparative evidence from selected EQ-i:YV studies

Study	Country / sample	Form and analysis	Principal evidence
Ferrándiz et al. (2012)	Spain; N = 1655 children and adolescents	Full form; exploratory factor/components analyses	Five-domain structure; α = 0.63–0.80; items 3 and 39 had weak item-total relations.
Sáinz et al. (2014)	Spain; N = 386 gifted youth, 11–18 years	Full form; exploratory analysis	Five components explained 41.1% of variance; reliability coefficients > 0.75.
Esnaola et al. (2017)	Spain; N = 484 adolescents, 12–18 years	Short form; longitudinal and cross-sectional comparisons	Little systematic age change; Stress Management changed among girls.
Navarro-Roldán et al. (2023)	Colombia; N = 1355, 8–14 years	Spanish short form; split EFA/CFA	Four-factor 21-item solution: CFI = 0.951, TLI = 0.944, RMSEA = 0.030; ω = 0.57–0.75.
Henning et al. (2026)	Canada; large school-based youth sample	30-item short form; CFA and invariance	Four correlated domains; acceptable fit, gender/education invariance, internal and test–retest reliability.
Present study	Portugal; N = 931, 8–16 years	54 substantive full-form items; ordinal CFA and target-ESEM	Target-ESEM replicated across split samples; domain α = 0.744–0.865 and ω = 0.760–0.866.

Note: Full- and short-form studies test related but non-identical item sets and latent structures. The table is a narrative comparison, not a meta-analysis. References are provided in the main manuscript.

Table S12. Average variance extracted and HTMT summary

Evidence	Domain / comparison	Estimate	Interpretation
AVE	Intrapersonal	0.396	Below 0.50
AVE	Interpersonal	0.384	Below 0.50
AVE	Stress Management	0.239	Below 0.50
AVE	Adaptability	0.346	Below 0.50
AVE	General Mood	0.377	Below 0.50
HTMT minimum	Stress Management–Adaptability	0.311	Below 0.85
HTMT maximum	Adaptability–General Mood	0.660	Below 0.85
HTMT range	All ten domain pairs	0.311–0.660	All below 0.85

Note: AVE = average variance extracted; HTMT = heterotrait–monotrait ratio of correlations. AVE was derived from standardized loadings in the ordinal five-factor CFA. HTMT was calculated from pairwise polychoric item correlations. The table reports the verified summary values available for the present analyses; HTMT values below 0.85 indicate that the domains are empirically distinguishable.
